# The Effect of the Frequently Used Cinacalcet for pHPT during the COVID-19 Pandemic on Perioperative Decrease in Parathyroid Hormone

**DOI:** 10.3390/jcm11072015

**Published:** 2022-04-04

**Authors:** Olga Radulova-Mauersberger, Julia Keßler, Ulrich Keßler, Katrin Stange, Sandra Korn, Jürgen Weitz, Ulrich Bork

**Affiliations:** 1 Department of Visceral, Thoracic and Vascular Surgery, University Hospital Carl Gustav Carus, Technische Universität Dresden, 01307 Dresden, Germany; olga.radulova-mauersberger@ukdd.de (O.R.-M.); julia.kessler@live.de (J.K.); sandra.korn@ukdd.de (S.K.); juergen.weitz@ukdd.de (J.W.); 2 Departments of Surgery and Endocrinology, Oberlausitz-Kliniken gGmbH and MVZ, 02625 Bautzen, Germany; ulrich.kessler@oberlausitz-kliniken.de (U.K.); katrin.spalteholz@gmail.com (K.S.); 3 National Center for Tumor Diseases (NCT/UCC), German Cancer Research Center (DKFZ), Faculty of Medicine and University Hospital Carl Gustav Carus, Technische Universität Dresden, Helmholtz-Zentrum Dresden—Rossendorf (HZDR), 01307 Dresden, Germany

**Keywords:** parathyroidectomy, pHPT, PTH, COVID-19, hypercalcemia, calcimimetic, cinacalcet

## Abstract

Background: Cinacalcet is a calcimimetic drug that has increasingly been used as a bridging therapy for primary hyperparathyroidism (pHPT), especially during the COVID-19 pandemic. The aim of our study was to investigate if preoperative cinacalcet therapy affects intraoperative parathyroid hormone (IOPTH) monitoring during parathyroidectomy, which is an important indicator for the success of surgery. Methods: In this single-center retrospective analysis, we studied the outcomes of 72 patients who underwent surgery for pHPT. We evaluated two groups: those with cinacalcet therapy before operation—the cinacalcet group (CG)—and those without medical therapy preoperatively (non-CG). In order to perform a between-group comparison of time trends, we fit a linear mixed-effects model with PTH as the response variable and predictors PTH levels preoperatively, group (cinacalcet yes/no), time, the group-by-time interaction, and a random intercept (per subject). Results: Our cohort included 51 (71%) women and 21 (29%) men, who were operated upon for pHPT in the period from January 2018 until August 2021. All patients were diagnosed with pHPT and 54% of the cohort were symptomatic for hypercalcemia. Moreover, 30% of the patients were treated with cinacalcet as a bridging therapy preoperatively, and this increased during the COVID-19 pandemic, as 64% of this group were treated in the last two years. Calcium values were significantly different before (*p* < 0.001) and after (*p* = 0.0089) surgery, but calcium level change did not differ significantly between the CG and non-CG. Parathyroid hormone (PTH) levels dropped significantly in both groups during 10 min IOPTH monitoring (*p* < 0.001), but there was no significant difference between the two groups (*p* = 0.212). Conclusions: In the examined patient cohort, the use of cinacalcet did not affect the value of IOPTH monitoring during surgery for pHPT.

## 1. Introduction

Primary hyperparathyroidism is the third most frequently diagnosed endocrinological disorder [1,2]. The annual incidence of pHPT is about 20–25 per 100,000, making it the most common cause of hypercalcemia [3]. Women are three to four times more likely to be affected than men, and the prevalence rises with increasing age [4,5,6]. pHPT results from excessive release of PTH from the parathyroid glands and is caused in 85–90% of cases by adenoma [7]. The clinical presentation of pHPT includes the typical signs for hypercalcemia with recurrent nephrolithiasis and fragility fractures due to renal calcium reabsorption and osteoclast-mediated bone resorption. Due to advances in paraclinical diagnostics, pHPT is now often diagnosed as an asymptomatic disease. The diagnosis is confirmed in patients with elevated serum calcium and PTH levels. Generally, if classical symptoms, such as nephrolithiasis, limb pain, and upper gastrointestinal tract ulceration, are lacking, but a typical biochemical constellation is detected, patients are described as asymptomatic. However, pHPT is often associated with non-specific symptoms, such as fatigue, loss of appetite, depression, constipation, or polyuria, which are not always recognized and reported by patients. Therefore, the definition of asymptomatic pHPT is somewhat controversial, and these patients should also be offered a surgical therapy [8].

Surgery is the only curative treatment for biochemically confirmed pHPT. It ensures a long-term and efficient therapy and is, therefore, considered to be indicated in case of biochemically confirmed pHPT [9,10]. Whether surgical treatment is performed depends on the patient’s condition and the informed agreement to surgery after a detailed explanation of the risks and complications associated with the procedure. Until now, it was considered that parathyroid hormone levels would drop to about 50% of the original baseline value measured 10 min after parathyroid adenoma excision [11].

For postponed surgery or as bridging option until operation, cinacalcet is being now increasingly applied. Cinacalcet, as a calcimimetic drug, increases the sensitivity of the calcium receptor in the parathyroid gland for extracellular calcium, lowers the production of PTH, and indirectly reduces serum calcium [12]. As a result, PTH and consecutive calcium levels in serum decrease. In the case of persistent calcium values above 2.8 mmol/L (11.23 mg/dL) with depleted fluid intake and symptomatic patients, cinacalcet is essential when surgery is delayed to avoid arrhythmia and a parathyroid crisis [12]. However, with the administration of cinacalcet, a lower level of parathyroid hormone is observed preoperatively, and accordingly, a smaller decrease is expected after surgery. Therefore, in patients who received cinacalcet therapy before undergoing surgery for pHPT, intraoperative parathyroid testing may not be as valid as in patients without such preoperative medical therapy. This hypothesis was tested in this study.

## 2. Materials and Methods

### 2.1. Study Design and Patient Data Acquisition

The study protocol was approved by the local ethics committee (IRB00001473) of the Technische Universität (TU) Dresden (decision number BO-EK-422082021). Data from all patients who underwent a resection of the parathyroid adenoma from January 2018 to August 2021 were retrospectively collected and analyzed in an electronic database. All procedures were carried out at the Department of Surgery of the District Hospital, Bautzen. Demographic data, operative details, and perioperative data, including blood results pre- and postoperatively, were collected. Comorbidity was recorded according to the ASA classification. For intraoperative monitoring, the parathyroid hormone was measured in blood samples taken at the beginning of the operation before skin incision, ten and twenty minutes after extirpation of the parathyroid adenoma. If PTH dropped >50% from the baseline value, the operation was successfully completed and another PTH value was measured 24 h after surgery.

Postoperative complications were reported according to the Clavien-Dindo classification (CDC) and were defined as any complication occurring after index surgery [13]. pHPT has been defined as hypercalcemia (reference values: 2.2–2.55 mmol/L (8.8–10.23 mg/dL)) and elevated PTH (reference: 14.5–87.1 ng/L).

The primary endpoint of the study was to investigate whether there are differences in intraoperative PTH dynamics between the two groups, CG and non-CG.

Secondary endpoints were the analysis of calcium level change and short-term patient outcome in both groups.

### 2.2. Statistical Analysis

In order to perform a between-group comparison of the time trend, we fit a linear mixed-effects model with PTH as the response variable and predictors PTH preoperative, group (cinacalcet yes/no), time, the group-by-time interaction, and a random intercept (per subject). A post hoc *t*-test was used to analyze the difference between the two groups at each time point separately. Descriptive statistics and univariate analysis using a Wilcoxon rank-sum test, ANOVA, and Fisher exact test were performed. A *p*-value of <0.05 was considered to be the threshold of statistical significance for all analyses. During the analyses, missing data were treated as missing completely at random. Data for PTH 24 h postoperatively were missing in 4 cases in the CG and 16 cases in the non-CG. Thus, a complete case analysis was performed, and some patients were excluded from the analysis. Statistical analysis was performed using R version 4.0.2 (The R Foundation for Statistical Computing, Vienna, Austria).

## 3. Result

### 3.1. Baseline Patient Characteristics

**Figure 1:**
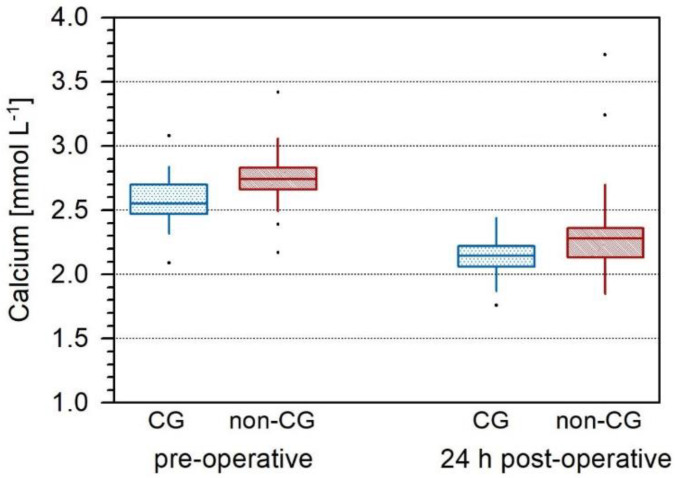
Box plot illustrating the time trends for calcium (mmol/L). Calcium values differ significantly in both groups, CG and non-CG (*p* = 0.001), and calcium decrease is significant pre- and postoperatively (*p* < 0.001) as well. However, calcium level change is similar and not significantly different between both groups.

Of the 72 patients included in the study, the mean age was 63.5 years (IQR: 53–76). The majority of patients were female (*n* = 51; 71%) (Table 1). All patients were diagnosed with pHPT, and 54% (*n* = 39) were symptomatic for hypercalcemia. Patients who were treated with cinacalcet preoperatively comprised 30% (*n* = 22) of the whole cohort and 55% of them were symptomatic. Calcium values were higher than the normal values in 45% of the cinacalcet group (median: 2.55 mmol/L (10.23 mg/dL); IQR 2.48–2.7) and 88% of the non-cinacalcet group (median: 2.74 (10.99 mg/dL); IQR: 2.66–2.83) preoperatively (Table 2). Values between the CG and non-CG groups were significantly different before (*p* < 0.001) and after (*p* = 0.0089) surgery, but no significant between-group differences in the calcium level change over time were found. Calcium values in the CG went down by 17% (compared to the initial value) and in non-CG by 16% (*p* = 0.673) on POD 1 (Figure 1).

### 3.2. Impact of Cincalcet on the Perioperative Biochemical Changes

**Figure 2:**
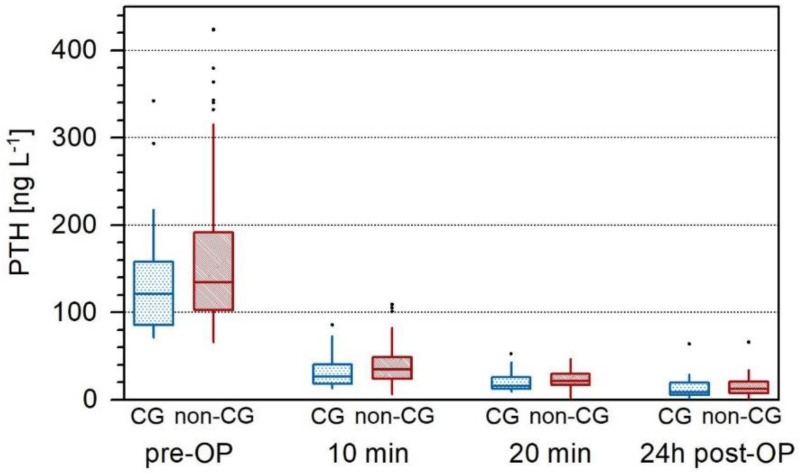
Box plot illustrating the time trends for PTH (ng/L). Time is significant (*p* < 0.001) as PTH drop was significant for both groups at any time point. Cinacalcet effect is not significant and not clinically relevant.

There was no significant difference found in the preoperative PTH value between both groups (*p* = 0.282). The CG group had slightly lower PTH levels (median: 121.35 ng/L; IQR 91.63–342.1) compared to non-CG (median: 134.7 ng/L; IQR 103.68–188.78). The difference between the average PTH levels in both treatment groups through all time points were not found to be significant. The PTH of patients with cinacalcet was, on average, 3.06 units lower than of those without (*p* = 0.348) over time. The differences between time points averaged through both groups were approximately 7.00 units. Additionally, 20 min after extirpation of the parathyroid adenoma, PTH was on average 13.84 units lower than at 10 min after extirpation (*p* < 0.001). The PTH 24 h after operation was, on average, 21 units lower than at 10 min with IOPTH monitoring (*p* < 0.001) and on average 7.13 units lower than at 20 min with IOPTH monitoring (*p* < 0.024). This indicates an overall decreasing trend in PTH over time. The interaction of group by time was found to not be statistically significant (*p* = 0.717). This indicates that cinacalcet has no relevant effect on IOPTH and data do not provide evidence showing a difference in time trends between the two treatment groups (Figure 2).

Surgery was always carried out as an extirpation of the parathyroid adenoma, and in 61% (*n* = 44) of the cases, a thyroid resection was synchronously performed. The median time for operation was 90 min (IQR: 35 min–115 min). An adenoma was intraoperatively not found to be localized as expected according to the preoperative diagnostic in 7% (*n* = 5) of the patients. Fresh frozen sections were, in five cases, negative for parathyroid tissue, and later confirmed to be parathyroid hyperplasia in the final pathology result. The median hospital stay was 6 (IQR: 5.0–6.0) days for both patient groups. There was no postoperative mortality and complications were recorded in nine (12%) cases, but no re-intervention was needed. Notably, the median number of operations performed annually was 22, and this decreased during the COVID-19 pandemic in 2021 to *n* = 9.

## 4. Discussion

Elective parathyroidectomy for pHPT has been frequently postponed in the era of the COVID-19 pandemic, and the European endocrinology guidelines proposed that cinacalcet should be considered as a bridging therapy while operation capacity is reduced [14]. However, calcimimetic therapy has increasingly been reported to be useful in improving preoperative biochemical parameters in pHPT even before the COVID-19 pandemic [12,15]. The drop in efficacy of this therapy, and sometimes its biochemical normalization, was reported in the results of a randomized controlled study and a systemic review [16,17].

Since PTH values are reduced under therapy with cinacalcet, the question arises as to whether PTH dynamics during the intraoperative monitoring are influenced as a result. Calcimimetic therapy was applied more commonly in our patient collective with the beginning of the COVID-19 pandemic, in 64% of the patients, which is in accordance with published evidence and the recommendation of the European Society for Endocrinology [12,14].

The main point of this study was to evaluate IOPTH monitoring regarding its decrease after adenoma extirpation by comparing patients with and without cinacalcet for pHPT before surgery. Furthermore, the changes in calcium levels in both groups and the short-term postoperative outcomes were analyzed.

We found that calcium values were significantly lower in the CG before and 24 h after operation. Notably, the preoperative therapy with cinacalcet did not significantly change the PTH values when comparing both groups. Only a slight reduction in preoperative PTH was observed in the CG group, but the values were above the norm for both groups. According to previous published studies evaluating the effect of cinacalcet preoperatively, PTH levels drop by between 8 and 15% in an average of 6 weeks of treatment, but remain elevated [16,18,19]. Although we observed similar results, our data on the duration of use of calcimimetics were too scarce to draw further conclusions.

When we analyzed PTH drop in both groups during IOPTH monitoring, we found no significant difference in between-group comparisons. IOPTH monitoring is evidently considered to be accurate with an excellent operative success, especially for pHPT, when preoperative image-guided diagnostics for the localization of the adenoma are implemented. The operative success then ranges between 97% and 99%, which makes PTH an important predictor for postoperative success and outcomes [20,21,22]. The guidelines from the German association of endocrine surgeons for the management of primary and renal hyperparathyroidism strongly recommends (consensus ++) the intraoperative PTH measurement for focused procedures when adenoma is preoperatively localized [7]. The time of IOPTH measurement is related to the dynamics of the marker. The monitoring helps surgeons to distinguish between parathyroid and non-parathyroid tissue and to confirm that parathyroid adenoma has been removed successfully [22]. It is important to follow a standard PTH sampling protocol.

**Figure 3:**
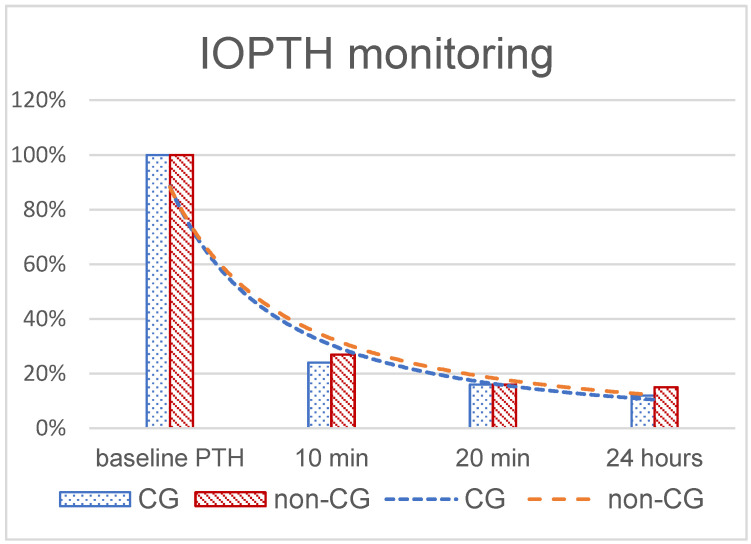
IOPTH monitoring of the PTH drop intraoperatively with trend lines for both groups, CG and non-CG.

In our cohort, a baseline value was obtained before incision and at 10 and 20 min after extirpating the parathyroid adenoma. According to the most widely used “Miami criterion”, if the PTH value drops 50% or more 10 min after the excision of the suspected gland, the procedure can be successfully terminated [11]. In line with this finding, our data showed a median of 74.5% decrease in PTH at 10 min after the adenoma for all patients with no statistically relevant difference between the cinacalcet and non-cinacalcet group (Figure 3). Some authors supposed that the PTH kinetics might be different if baseline levels are normal or just slightly elevated, as in the case of multi-gland diseases [23]. In our cohort, we had only one patient with a multi-gland disease, and in the five cases where parathyroid hyperplasia was found, we did not observe any difference in the PTH dynamics perioperatively. Clinical variables such as patient age, gland weight, serum calcium, vitamin D status, and renal function are reported to influence the IOPTH drop [2]. In our patient collective, we did not find any significant differences in age and renal function in the CG and non-CG. Preoperative and intraoperative PTH levels also did not differ statistically, but IOPTH drop was adequate at >50% and the calcium value decrease differed significantly after operation. In short-term follow-up, morbidity was 12%, and there was no case of postoperative mortality. The time for operation and hospital stay did not differ in both groups.

The strength of our study was to recognize if cinacalcet and its consecutive biochemical changes influence the IOPTH dynamics. This is a fundamental question regarding the increasing utilization of calcimimetics as bridging therapies during the COVID-19 pandemic, when access to parathyroidectomy is limited. In our cohort, we did not find a significant difference in the IOPTH dynamics. Further, we analyzed the impact on calcium level changes and on the short-term postoperative outcome and did not find any significant differences between both groups, although calcium values were pre- and postoperatively lower in the CG compared to the non-CG.

The most important limitation of this study is its retrospective design. We acknowledge that our conclusions are based on a report with a small sample size, especially in the CG, and are restricted to a patient load at a single institution. This might not reliably validate the dynamics of the analyzed parameters. It provides, however, a trend that should be investigated in further studies. To our knowledge, this is the first report that aims to examine the impact of cinacalcet on the IOPTH in pHPT.

## 5. Conclusions

In summary, we conclude that bridging therapies with calcimimetics does not influence the IOPTH monitoring and the level of PTH drop intraoperatively. Calcium levels are considerably lower and even normalized after cinacalcet therapy and drop further postoperatively, but they have no impact on morbidity and hospital stay. Cinacalcet may be used in cases of postponed surgery due to the COVID-19 pandemic without influencing the peri- and postoperative course for parathyroidectomy. Further studies with larger patient cohorts should be performed to confirm or disprove this conclusion.

## Figures and Tables

**Table 1:** Patients’ baseline characteristics.

	All Patients	Cinacalcet (CG)	Non-Cinacalcet (non-CG)
Number of Patients (*n*)	72	22	50
Age ^†^	63.5 (53–76)	60 (52–70)	66 (55–77)
Sex *			
Male	21 (29%)	11 (52%)	10 (48%)
Female	51 (71%)	11 (22%)	40 (78%)
ASA *			
I	10 (14%)	3 (14%)	7 (14%)
II	38 (53%)	12 (55%)	26 (52%)
III	23 (32%)	6 (8%)	17 (34%)
IV	1 (1%)	1 (4%)	0
Symptomatic pHPT *	39 (54%)	12 (55%)	27 (54%)
Asymptomatic pHPT *	33 (46%)	10 (45%)	23 (46%)
Chron. renal failure *	3 (4%)	1 (5%)	2 (4%)
Serum creatinine preoperative (45–84 µmol/L) ^†^	71.5 (59.7–82.2)	74.5 (62.2–89.2)	71 (59.0–80.7)
Serum urea preoperative (2,8–8,1 mmol/L) ^†^	4.6 (3.8–5.5)	4.5 (3.8–5.6)	4.6 (3.8–5.5)
Surgical procedure *			
NSD adenom extirpation	72 (100%)	22 (100%)	50 (100%)
Thyreoidectomy	23 (32%)	6 (27%)	17 (34%)
Hemithyreoidectomy	17 (24%)	5 (23%)	12 (24%)
Duration of surgery (min) ^†^	90 (59.5–115.0)	90 (62.5–100.7)	90 (56.2–115.0)
Hospital stay (days) ^†^	6 (5.0–6.0)	6 (5.2–6.0)	6 (5.0–6.7)

Data are presented as * *n* (%) or ^†^ median (interquartile range, IQR); pHPT: primary hyperparathyroidism; ASA: American Society of Anesthesiologists physical status classification system.

**Table 2:** Perioperative biochemical changes.

Laboratory Parameter (Normal Values)	CG	non-CG	*p* Value
Calcium preoperative (2.2–2.55 mmol/L) (8.8–10.23 mg/dL)	2.55 (2.48–2.7) mmol/L 10.23 (9.95–10.82) mg/dL	2.74 (2.66–2.83) mmol/L 10.99 (10.67–11.35) mg/dL	<0.001 *
Calcium 24 h postoperative (2.2–2.55 mmol/L) (8.8–10.23 mg/dL)	2.14 (2.06–2.21) 8.58 (8.26–8.86) mg/dL	2.28 (2.13–2.35) mmol/L 9.14 (8.54–9.42) mg/dL	0.008 *
PTH preoperative (14.5–87.1 ng/L)	121.3(91.6–157.0)	134.7 (103.7–188.8)	0.282 ^†^
PTH decrease of ≥50%			
PTH 10 min avarage value (min–max)	26.7 (18.6–40.0) 44.25 (12.9–85.6)	34.7 (23.8–48.5) 57.65 (6.3–109)	0.227 ^†^
PTH 20 min	15.9 (12.6–25.1)	21.4 (17.1–28.8)	0.790 ^†^
PTH 24 h	8.55 (5.55–18.3)	12.2 (7.15–20.37)	0.546 ^†^

Data are presented as median (interquartile range, IQR); for PTH, 10 min average values (minimum and maximum) are presented for each group; *p* * value in univariate analysis using Wilcoxon rank-sum test; *p* ^†^ value significance validated in post hoc *t*-test. Bold: significant value.

## Data Availability

The data are available from the corresponding author on request.

## Abbreviations

ASA	American Society of Anesthesiologists
COVID-19	coronavirus disease 2019
IQR	interquartile range
IOPTH	intraoperative parathyroid hormone
PTH	parathyroid hormone
pHPT	primary hyperparathyroidism
